# Parathyroidectomy Restores Muscle Strength and Transcriptome in Individuals With Primary Hyperparathyroidism

**DOI:** 10.1210/clinem/dgaf418

**Published:** 2025-07-22

**Authors:** Sofia Björnsdotter-Öberg, Anna Koman, Mikael Skorpil, Henric Rydén, Johanna T Lanner, Anna Krook, Inga-Lena Nilsson, Nicolas J Pillon, Carolina Nylén

**Affiliations:** Department of Molecular Medicine and Surgery, Karolinska Institutet, Stockholm SE-171 76, Sweden; Department of Molecular Medicine and Surgery, Karolinska Institutet, Stockholm SE-171 76, Sweden; Department of Breast, Endocrine Tumors and Sarcoma, Karolinska University Hospital, Stockholm SE-171 64, Sweden; Department of Molecular Medicine and Surgery, Karolinska Institutet, Stockholm SE-171 76, Sweden; Department of Neuroradiology, Karolinska University Hospital, Stockholm SE 171 76, Sweden; Department of Neuroradiology, Karolinska University Hospital, Stockholm SE 171 76, Sweden; Department of Clinical Neuroscience, Karolinska Institutet, Stockholm SE 171 76, Sweden; Department of Physiology and Pharmacology, Biomedicum, Karolinska Institutet, Stockholm 171 77, Sweden; Department of Physiology and Pharmacology, Biomedicum, Karolinska Institutet, Stockholm 171 77, Sweden; Department of Molecular Medicine and Surgery, Karolinska Institutet, Stockholm SE-171 76, Sweden; Department of Breast, Endocrine Tumors and Sarcoma, Karolinska University Hospital, Stockholm SE-171 64, Sweden; Department of Physiology and Pharmacology, Biomedicum, Karolinska Institutet, Stockholm 171 77, Sweden; Department of Molecular Medicine and Surgery, Karolinska Institutet, Stockholm SE-171 76, Sweden; Department of Breast, Endocrine Tumors and Sarcoma, Karolinska University Hospital, Stockholm SE-171 64, Sweden

**Keywords:** parathyroidectomy, primary hyperparathyroidism, muscle strength

## Abstract

**Context:**

Primary hyperparathyroidism leads to hypercalcemia and muscle dysfunction. Muscle weakness is associated with increased morbidity and mortality but is overlooked in surgical treatment guidelines. While parathyroidectomy is the only curative treatment, its effects on skeletal muscle strength and molecular remodeling remain underexplored.

**Objective:**

Determining functional and molecular changes in skeletal muscle before and after parathyroidectomy.

**Methods:**

A prospective observational study was conducted in the spring and fall of 2023. Patients underwent surgery at the Endocrine and Sarcoma unit at the Karolinska University Hospital in Stockholm, Sweden. A total of 21 postmenopausal women with primary hyperparathyroidism planned for surgery were included, whereof 15 completed the study protocol. Participants had no disabling comorbidities. Muscle function tests, muscle biopsies, MRI, and biochemical panels were analyzed before and after parathyroidectomy. Muscle composition of *m. vastus lateralis* was tested with MRI and transcriptomic analysis of muscle biopsies. Leg strength was evaluated with timed stands test and peak torque tests. Activity level was estimated from questionnaires.

**Results:**

Parathyroidectomy normalized calcium levels (*P* < .001) and improved muscle strength (*P* < .005). Muscle volume increased (*P* = .023) and fat fraction was reduced (*P* = .013), without changes in physical activity levels. Transcriptomic analysis identified 981 differentially expressed genes postsurgery, enriched in pathways mirroring exercise-induced adaptations.

**Conclusion:**

These findings highlight the impact of parathyroidectomy on skeletal muscle function and suggest that muscle assessments should be included in surgical referral criteria to address age-related muscle decline and improve long-term outcomes.

Primary hyperparathyroidism (pHPT) is the third most common endocrine disorder following diabetes and hypothyroidism (1). pHPT is increasingly common with older age and up to 5% of postmenopausal women are suggested to be affected (2). The prevalence of pHPT has risen over recent decades due to increased biochemical screening but also due to an aging population (3). This rising prevalence poses a major public health concern, as untreated pHPT can lead to increased morbidity leading to higher dependency on healthcare services (4).

The effects of parathyroid hormone (PTH) on bone metabolism are well established and the presence of osteoporosis is the main reason for surgical intervention with parathyroidectomy, ultimately to prevent fractures. The effects of PTH on skeletal muscle are less studied; however, muscle weakness is regarded as a common symptom of pHPT (5). Several clinical studies have shown that patients with pHPT are weaker than the age-matched population (6, 7). Impaired muscle strength and endurance are independent predictors of prolonged hospitalization and readmission after acute illness, disability, and mortality (8). In the aging population, decreased muscle volume and impaired muscle strength are associated with risks of falls, fractures, and related morbidity and mortality (9). Consequently, preserving functional capacity in the older adults remains a critical public health challenge. Previous clinical studies have investigated and confirmed the restorative function of curative parathyroidectomy on muscle effect in grip strength, postural stability, and strength of the upper and lower extremities (6, 10-12).

Given that muscle weakness is a common symptom of pHPT, we aimed to more deeply investigate the clinical and molecular effects of parathyroidectomy on muscle function. Our findings contribute to the scientific foundation for future considerations on patient selection for surgical intervention.

## Methods

### Study Design

The study was a prospective observational study, in which the outcomes were analyzed before and 3 months after surgery for pHPT. Each subject served as their own control.

### Study Population

A total of 21 postmenopausal women with pHPT, referred for surgical assessment in accordance with accepted international guidelines (13), were recruited from the Department of Endocrine Surgery at the Karolinska University Hospital in Solna, Stockholm, Sweden, between October 2022 and February 2023. Only postmenopausal women were included, to control for sex hormone differences. Twenty women were admitted from primary care units, and one from the urology department. Of these, 8 participants had osteoporosis verified by bone density measurements and another 9 had osteopenia. Fatigue was reported in 14 cases, muscle weakness or pain in 8, and psychiatric symptoms such as memory loss or depression in 10 participants.

At inclusion, all study participants had biochemically verified pHPT with elevated ionized calcium and elevated or inappropriately elevated PTH. Participants lived independently, had no restrictions on activities of daily living, and were able to move without aid. Exclusion criteria were kidney deficiency or disease, treatment with anticoagulant medications, antidiabetic treatment, anti-inflammatory or immune-modulating medication, lithium, or thiazide diuretics. All participants provided written informed consent. The protocol was in accordance with the Helsinki declaration, version 2013, and was approved by the Regional Ethical Review Board, Stockholm, Sweden.

Six women withdrew from the study at different stages: 1 relocated, 2 withdrew for personal reasons, 1 due to a psychological condition, and 2 due to discomfort from the muscle biopsy. One participant was found to have persistent disease at the 6-week postoperative follow-up and subsequently underwent reoperation 2 months after the initial surgery.

### Parathyroid Surgery

Parathyroidectomies were performed by experienced parathyroid surgeons at the Department of Breast, Endocrine Tumours and Sarcoma at the Karolinska University Hospital. Parathyroid adenomas were preoperatively localized with ultrasound and/or Tc99 Sestamibi-scintigraphy/computed tomography (CT). The patients were operated on by a focused approach under general anesthesia. PTH measurements were performed directly postoperatively, and an adequate decline was regarded as cure. The patients were discharged the same afternoon or the following day depending on their social and housing situation. All patients had histopathological proven adenomas and were normocalcemic at the 3-month follow-up.

### Clinical Chemistry

Anthropometric measures (weight, length, and BMI) and blood samples for clinical chemistry were collected the morning of the surgery. Clinical chemistry measurements were repeated 3 months postsurgery. All biochemical analyses were performed using routine methods by the Karolinska University Laboratory in Stockholm.

Plasma concentrations of bio-intact PTH were determined with electrochemiluminescence immunoassay on the Elecsys PTH(1-84) (gen 3) system (Roche Cat# 07027745190, RRID:AB_2895650, Roche Diagnostics GmbH, Mannheim, Germany). Serum concentrations of 25-hydroxyvitamin D were measured by chemiluminescence with Elecsys Vitamin D total III assay (gen 3) (Roche Cat# 09038078, RRID:AB_2909604, Roche Diagnostics GmbH, Mannheim, Germany). Bone-specific alkaline phosphatase (ALP) was measured using sandwich enzyme immunoassay ELISA (mouse monoclonal antibodies) with BAP OSTASE (DiaSorin Cat#310970, RRID:AB_3696965, DiaSorin, Saluggia, Italy) on LIAISON XL system (LIAISON, Brighton, MA, USA).

### Activity Level

Physical activity level was assessed with a standardized questionnaire from the Swedish National Board of Health and Welfare on time spent on pulse-increasing activity such as jogging, ball sports, or aerobics; activities on daily life such as gardening and strolling; and sedentary time (14). Participants filled in the questionnaire 1 week before and 3 months after parathyroidectomy.

### Skeletal Muscle Strength

Lower body strength was measured with the *timed stands test*. A 44.5-cm high stool was used for the test. The time required for participants to complete 10 full stands from a sitting position was recorded with a stopwatch and rounded to the nearest tenth of a second. Participants received verbal test instructions before the test, were instructed to perform the test either barefoot or with a low-heeled shoe, and not to use upper extremities during the test. Muscle strength was tested 1 week before surgery and then again 3 months after surgery. Improvement in muscle strength is indicated by a shorter time to perform the test.

Maximal isokinetic strength of the quadriceps was measured to determine isokinetic peak torque values. The protocol was performed on the left thigh using a Biodex System 4 Pro dynamometer (Biodex Medical System Inc, Shirley, NY, USA) 1 week before and 3 months after surgery. Subjects performed a 5-minute warmup on a stationary bike before running the protocol. The test chair was adjusted to put subjects in a seated, upright position, with 85° hip flexion. The range of motion tested was 180°, with the left lateral femoral epicondyle positioned in line with the dynamometer shaft. The subject was secured with Velcro straps around the left wrist, the hip, and the trunk, and was instructed not to use the hand levers during the exercise. After gravity correction, the range of motion limits were specified as 180° from the subjects' full knee extension to a complete knee flexion. Before the protocol was run, the subjects were given verbal test instructions and allowed to make 3 practice attempts to familiarize themselves with the dynamometer.

The protocol tested concentric strength of the quadriceps at 2 angular velocities. Subjects did 6 repetitions in the concentric-concentric mode at each level, with 3 minutes of rest between velocity levels. Peak torque was registered at each level.

### Magnetic Resonance Imaging

Magnetic resonance imaging (MRI) was used to assess changes in muscle composition and volume before and after surgery. MRI scans of the left thigh were performed at the Karolinska University Hospital 1 week before and 3 months after parathyroidectomy in all participants except 1, in whom the right thigh was depicted due to a left-sided hip prothesis.

Segmentation and analysis of muscle composition and volume of the anterior compartment (*m. sartorius* and *mm. quadriceps femoris*) was performed in 3D Slicer Software version 5.6.1. Acquisitions were blinded to the analyzer, and 3 analyses of each acquisition were performed. Presurgery and postsurgery data were only matched after the analysis was completed.

Study participants were examined on a 3-T GE Signa Premier MR system with a 30-channel flexible phased array coil (AIR Coil, GE Healthcare, Milwaukee, WI, USA). The left thigh musculature was entirely covered with 2 stacks of 3D spoiled gradient 5 echo Dixon sequence generating proton density fat fraction maps, in-phase, out-of-phase, fat, R2*, and water images. Relevant imaging parameters were: TE1/ΔTE = 1.4/0.7 ms. TR = 13.6 ms, flip angle = 5, matrix = 256 × 180 × 70, FOV = 300 × 210 mm, in-plane resolution = 1.2 × 1.2 mm, slice thickness = 5 mm and number of averages (NSA) = 1. The Dixon MRI method provides high repeatability in measuring muscle volume and fat fraction (15).

### Muscle Biopsies

Muscle biopsies were collected with the *Bergström muscle biopsy technique* (16) in local anesthesia (Marcain® with adrenaline 5 mg/mL, Aspen Nordic, Ballerup, Denmark) from *m. vastus lateralis* of the left thigh concomitant with the surgical procedure, before the subject was exposed to general anesthesia. The study participants were fasted at least 6 hours before the muscle biopsy procedure. All biopsies were collected in the morning. Follow-up muscle biopsies were obtained with the same method 3 months after surgery. Muscle biopsies were immediately frozen in liquid nitrogen and stored at −80 °C. Five participants experienced discomfort or pain from the biopsy procedure and 3 participants had small hematomas after the procedure.

### RNA Sequencing

Total RNA from skeletal muscle biopsies was isolated using E.Z.N.A. Total RNA Kit I, #R6834-02, Omega Bio-Tek, USA, and treated with E.Z.N.A. Tissue DNA kit, #D3396-02, Omega Bio-Tek, USA. The biopsies were analyzed at the BEA Core Facility at Karolinska University Hospital in Huddinge. Libraries were generated using TruSeq Stranded Total RNA (Illumina, San Diego, California, United States) and sequenced on an Illumina Novaseq 6000 Sequencing System (Illumina). Quality and adapter trimming of reads was performed using Cutadapt (v3.5). Sample quality was assessed using FastQC (v0.11.8) and MultiQC (v1.14). Reads were aligned to the Ensembl GRCh38/GRCm38 reference genome using STAR (v2.7.9d). Counts for each gene were obtained using featureCounts (v1.5.1). Analysis of RNA sequencing was performed in R v4.4.1. Differential expression analysis was performed with the edgeR package. The logCPM (count per million) values for each gene were calculated using limma's *voom* function. Relationships between genes were established using StringDB (17).

Genes with false discovery rate (FDR) < 0.05 were considered differentially expressed, without imposing a fold-change threshold to preserve the detection of subtle but biologically meaningful regulatory changes. Gene set enrichment analysis was performed using clusterProfiler (18) on genes ranked on fold-change using the Gene Ontology database for pathway annotation. Motif enrichment analysis on genes with FDR < 0.05 was performed using Enrichr based on the ENCODE and ChEA Consensus transcription factors from ChIP-X (19).

To compare transcriptomic responses with exercise training, we used preprocessed data from the MetaMEx database (20). Data from the MetaMEx database was matched to the present cohort by extracting data from healthy female participants over 52 years of age who underwent chronic aerobic training for at least 2 weeks. Gene-level comparisons were performed by matching symbols across datasets, and correlation analyses were based on log fold changes rather than *P* values, due to differences in sample sizes and statistical power. Directional concordance in gene expression was used to infer shared transcriptional signatures between parathyroidectomy and exercise training.

### Statistical Analysis

Normality of the data was assessed using the Shapiro-Wilks test. For normally distributed data, paired *t* tests were used to compare pre- and post-intervention measurements. For non-normally distributed data, the Wilcoxon matched-pairs signed rank test was applied. The SD was calculated for each parameter. Results are presented as mean differences and SD. Statistical analysis were performed using GraphPad Prism Software version 10, and a significance threshold of *P* < .05 was used for all tests.

## Results

### Normalization of Biochemical Parameters 3 Months After Parathyroidectomy

The mean age of study participants at baseline was 68 ± 9.8 years and mean BMI 27.0 ± 4.9. Parathyroidectomy effectively cured pHPT as evidenced by the normalization of calcium and related plasma markers (Table 1). Both ionized and total calcium levels decreased postoperatively (*P* < .001), returning to within normal physiological ranges for all but 1 participant, who had mildly elevated ionized calcium at follow-up (1.35 mmol/L). PTH levels showed a marked decrease following surgery (*P* = .013), although at 3 months, 9 participants still had mildly elevated PTH levels. Serum 25-hydroxyvitamin D and serum phosphate levels increased (*P* < .001), whereas alkaline phosphatase decreased postoperatively. While no significant change was observed in serum albumin (*P* = .572), creatinine levels increased postoperatively (*P* = .003), and the estimated creatinine clearance decreased (*P* = .006). Taken together, these findings provide biochemical evidence that parathyroidectomy normalized parathyroid function and improved control of hypercalcemia and calcium-phosphate balance.

**Table 1.** Changes in biochemical measures 3 months after curative parathyroidectomy

	Before	After	Normal range	*P* value
**Ionized calcium, mmol/L**	1.39 ± 0.05	1.26 ± 0.04	1.15-1.33	<.001
**Calcium, mmol/L**	2.60 ± 0.09	2.33 ± 0.09	2.15-2.50	<.001
**Parathyroid hormone, pmol/L**	10.23 ± 4.49	7.49 ± 2.57	1.60-6.00	.013
**Phosphate, mmol/L**	0.85 ± 0.12	0.92 ± 0.13	0.80-1.50	.011
**Albumin, g/L**	36.95 ± 2.48	37.65 ± 2.42	36-45	.572
**Creatinine, μmol/L**	64.30 ± 10.86	73.41 ± 11.14	<90	.003
**Creatinine clearance, mL/min/1.73 m^2^**	71.61 ± 8.68	66.47 ± 11.12	>60	.006
**25-OH-vitamin D, nmol/L**	65.86 ± 12.62	82.18 ± 15.39	50-250	<.001
**Bone alkaline phosphatase, µkat/L**	19.11 ± 8.45	14.37 ± 5.87	0.70-1.90	.005

Data represents mean ± SD. Wilcoxon matched pair signed rank test, *n* = 17. Normal references from the Karolinska University Hospital Laboratory.

### Improved Skeletal Muscle Strength Following Parathyroidectomy

**Figure 1.**
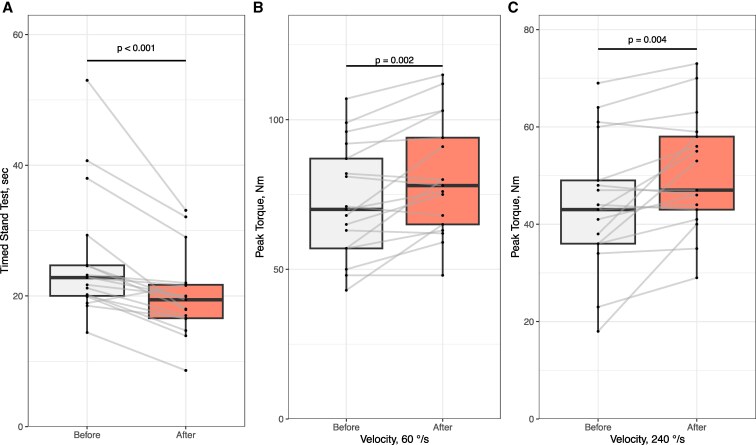
Improved lower extremity strength. A, Timed stand test (sec) before and 3 months after surgery, *n* = 17. Wilcoxon Signed Rank Test, 2-tailed significance level *P* < .05. B & C, Peak torque of quadriceps muscles of the left thigh at 60 (B) and 240 (C) degrees per second before and 3 months after surgery, *n* = 17. Student paired *t* test, 2-tailed significance level *P* < .05. Boxes A-C show median and interquartile range, whiskers indicate the range excluding outliers.

Muscle function, as assessed by the timed stands test, showed normalization 3 months after parathyroidectomy (Fig. 1A). The test is a standardized assessment of lower limb strength, and the mean times to perform the test range from 16.8 to 20.9 seconds in healthy individuals 55 to 85 years of age, with an upper healthy limit of 26.1 seconds in the oldest age group (21). At baseline, 9 of the 21 participants performed worse than their age-specific normal range. Three months after surgery, 14 out of 17 participants performed within their age-specific normal range. Prior to surgery, participants required an average of 25.5 ± 9.7 seconds to complete the test (Fig. 1A). After surgery, this time decreased to 20.1 ± 6.3 seconds (*P* < .001), indicating enhanced lower limb strength and functional mobility following the normalization of calcium levels.

Isokinetic testing was used to evaluate the function of the quadriceps muscles. An angular speed of 60°/s was used to reflect maximal strength of the muscle, while 240°/s was used to evaluate endurance. At 60°/s, mean peak torque in the quadriceps muscles improved from 72.7 ± 19.1 Newton meters (Nm) to 80.7 ± 19.5 Nm (Fig. 1B, *P* = .002). At 240°/s, mean peak torque improved from 44.4 ± 13.7 Nm to 50.5 ± 11.9 Nm (Fig. 1C, *P* = .004). Muscle function at baseline was analyzed in relation to ionized calcium levels; however, no correlations were found (data not shown). Altogether, all 17 participants showed profound increases in both maximal strength and endurance of the proximal leg following parathyroidectomy.

### Increased Muscle Volume Without Changes in Activity Level Following Parathyroidectomy

**Figure 2.**
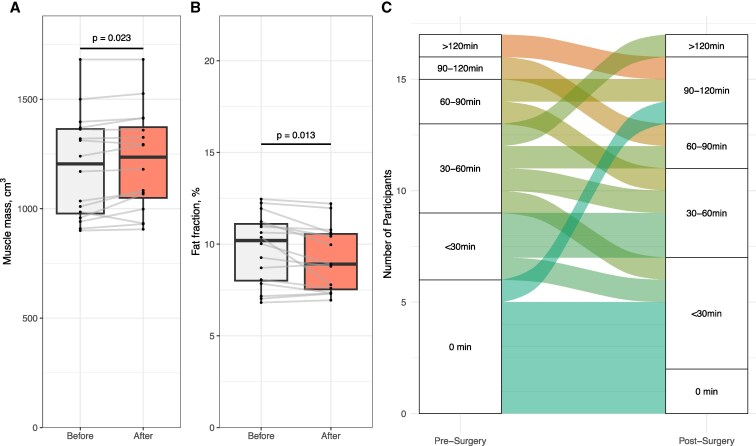
Increased muscle composition without change in weekly time spent on pulse-increasing physical activity. A & B, MRI-measured muscle volume (cm^3^) (A) and fat fraction (%) (B) in the anterior compartment of the left thigh before and 3 months after parathyroidectomy, *n* = 16. Student paired *t* test, 2-tailed significance level *P* < .05. Boxes A & B show median and interquartile range, whiskers indicate the range excluding outliers. C, Responses to FASTA-questionnaire rating self-evaluated weekly time spent on pulse-increasing physical activity before and 3 months after surgery, *n* = 17.

An increase in muscle volume was observed in MRIs, a standardized method to associate frailty and muscle strength (15, 22), in 16 participants 3 months after parathyroidectomy (Fig. 2A). Prior to surgery, the mean muscle volume of the anterior compartment of the left thigh was 1193 ± 239 cm^3^. After surgery, the muscle volume increased to 1217 ± 231 cm^3^ (*P* = .023). While muscle volume increased, fat fraction showed a marked decrease, from 9.7% ± 1.9% to 9.2% ± 1.8% (*P* = .013) after surgery (Fig. 2B). Patients reported no changes in pulse-increasing physical activity level in self-reported questionnaires 3 months after surgery compared to before surgery (Fig. 2C, *P* = .156, *n* = 17). Altogether, our analysis revealed changes in muscle composition following surgery, independent of changes in level of recreational exercise.

### Transcriptional Remodeling in Response to Parathyroidectomy in Skeletal Muscle

**Figure 3.**
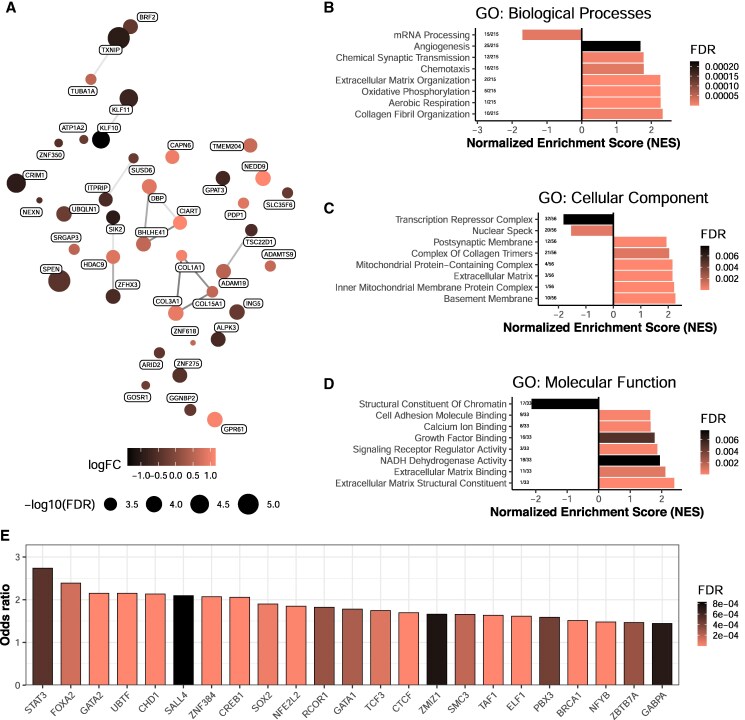
Remodeling of skeletal muscle transcriptome following parathyroidectomy. A, Top upregulated and downregulated genes (FDR < 0.001) clustered according to known relationships between proteins from StringDB. B-D, Gene Set Enrichment analysis on genes ranked on fold-change using the Gene Ontology dataset for biological processes, cellular component, and molecular function. E, Top transcription factor (FDR < 0.001) regulated by parathyroidectomy.

Increased circulating creatinine levels, along with improved muscle strength and mass, provide evidence for skeletal muscle remodeling following parathyroidectomy. Transcriptomic analysis of skeletal muscle tissue identified 981 mRNAs that were differentially expressed after surgery compared to presurgery levels (FDR < 0.05). These genes grouped into various clusters, suggesting the activation of diverse pathways although none of the genes identified are typically associated with skeletal muscle function (Fig. 3A). Gene Ontology enrichment analysis of biological processes showed that genes altered after parathyroidectomy were linked to angiogenesis, extracellular matrix organization, and mitochondrial respiration (Fig. 3B). The differentially expressed genes were also connected to transcriptional machinery, mitochondrial complexes, and collagen formation (Fig. 3C). Molecular functions associated with these gene expression changes included calcium homeostasis, transcriptional regulation, growth pathways, extracellular matrix remodeling, and oxidative metabolism (Fig. 3D). Motif enrichment analysis on the promoter regions of significantly regulated mRNAs (FDR < 0.05) highlighted several transcription factors involved in the gene expression changes following surgery (Fig. 3E), notably STAT3 (signal transducer and activator of transcription 3) and CREB1 (cAMP responsive element binding protein 1), transcription factors that regulate inflammation, metabolism, and stress responses, which are critical in the context of metabolic diseases.

### Training-Like Transcriptomic Response to Parathyroidectomy in Skeletal Muscle

**Figure 4.**
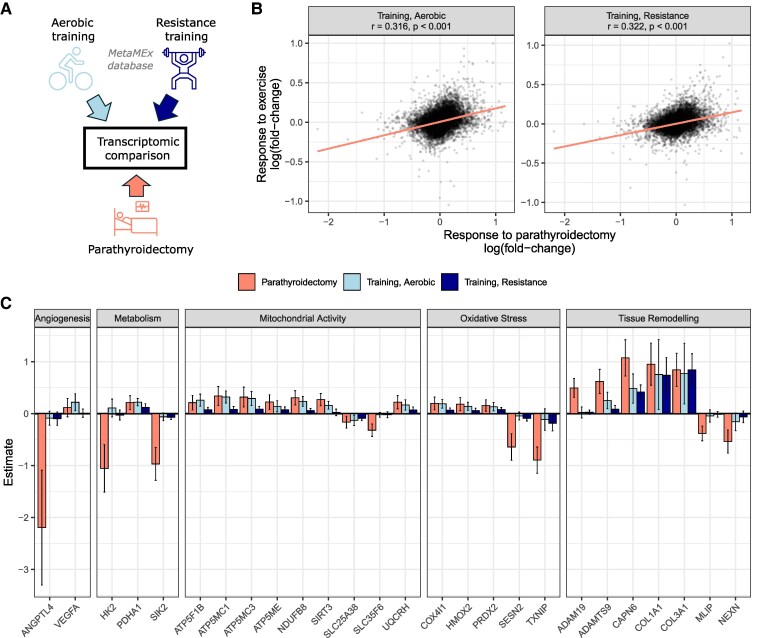
Parathyroidectomy recapitulates the transcriptomic response to exercise training in skeletal muscle. A, Schematic representation of the comparative transcriptomic analysis. B, Spearman correlation of the transcriptomic response to parathyroidectomy with mRNA changed induced by aerobic and resistance exercise training. C, Selected genes involved in skeletal muscle remodeling significantly changed by parathyroidectomy and exercise training.

The transcriptomic response to parathyroidectomy was compared to typical mRNA profiles from exercise training or inactivity using the MetaMEx database (20). Despite no changes in activity levels (Fig. 2), parathyroidectomy induced mRNA alterations that closely resembled the transcriptomic response to exercise training, with strong correlations of mRNA changes between parathyroidectomy and both resistance and aerobic training (Fig. 4B). By looking at selected genes in our transcriptomic analyses, we observed that genes involved in angiogenesis like *VEGFA* (vascular endothelial growth factor a) and in the regulation of lipid metabolism, like *ANGPTL4* (angiopoietin-like 4) (23) were changed in the same direction by parathyroidectomy and exercise training (Fig. 4C). Parathyroidectomy induced changes in genes involved in metabolism and mitochondrial activity that were consistent with exercise training, suggesting that the surgery had beneficial effects on skeletal muscle mitochondrial biogenesis and metabolism. For example, *HK2* (hexokinase 2), a gene that is involved in mitochondrial function and regulation of the rate-limiting step of glucose metabolism (24), was decreased, while genes encoding collagen with association to tissue remodeling like *COL1A1* and *COL3A1*, increased (Fig. 4C) (25, 26). Like exercise training, parathyroidectomy also induced changes in antioxidant pathways and extracellular matrix genes, consistent with an increased skeletal muscle strength. Altogether, the transcriptomic analysis revealed that, in the absence of changes in physical activity levels, parathyroidectomy activates potent pathways leading to remodeling of skeletal muscle recapitulating the response to exercise training.

## Discussion

In this comprehensive study on the clinical and molecular effects of pHPT, we demonstrate improvements in muscle strength following parathyroidectomy, accompanied by radiology-verified changes of the muscle composition and RNA expression changes in muscle biopsies. The changes after surgery resemble those observed after resistance and endurance exercise, despite no substantial changes in physical activity level.

Our findings show that parathyroidectomy results in up to 20% improvements in muscle strength in postmenopausal women. Patients with pHPT present with markedly reduced muscle strength in both the lower and upper limbs compared to healthy controls (6). Several previous studies have demonstrated skeletal muscle strength improvements after surgery, in line with our results. Parathyroidectomy improves health-related quality of life (27), grip strength (11), proximal muscle strength (28), and functional capacity (29). Nevertheless, despite evidence of clinical improvements in muscle strength following surgery, muscular symptoms have been overlooked in guidelines and recommendations for the treatment of pHPT. Current European criteria for surgery in patients with pHPT are primarily based on quantitative evidence of hypercalcemia, as well as bone- and kidney involvement (30). In contrast, the American guidelines offer only a limited recommendation to consider muscle weakness when making decisions about surgery (31). Restoring muscle strength after parathyroidectomy can contribute to healthy aging and alleviate the burden on the healthcare system. Muscle function impairments are frequently regarded as a normal part of the aging process and are not always recognized by patients as symptoms of disease. In our cohort, although most participants did not identify muscle weakness as a primary symptom prior to surgery, the improvement in muscle function following surgery was clearly evident.

Our results indicate that the volume of the thigh muscle increases 3 months after curative parathyroidectomy, despite no change in activity levels. Previous studies of skeletal muscle biopsies in patients with pHPT have shown muscle atrophy and changes in gene expression of muscle regulatory genes (32, 33). In vitro studies have verified the presence of PTH receptors on human muscle cells (34), and the direct involvement of PTH on the myocyte (35). Although hypercalcemia can cause muscle weakness (36), short-term medical normalization of hypercalcemia was effective in predicting the effect on muscle strength after parathyroidectomy but with a relatively low negative predictive value, indicating that the muscle weakness seen in pHPT is not linked to hypercalcemia alone (37). Although the clinical improvements of skeletal muscle strength after parathyroidectomy are well established, the mechanisms underlying improvements in skeletal muscle strength need further investigation.

Gene set enrichment analysis using the Gene Ontology and Reactome databases revealed that genes altered by surgery in this cohort are involved in pathways related to extracellular matrix remodeling, calcium ion binding, and mitochondrial protein-containing complex, highlighting a molecular link between parathyroidectomy and skeletal muscle structure and function. Furthermore, the transcriptomic profile observed indicates a substantial tissue remodeling, including both structural and metabolic components in skeletal muscle, with effects resembling those seen after resistance and aerobic exercise (20). We identified several transcription factors involved in the gene expression changes following surgery. Notably, UBTF and CREB1 show strong enrichment. UBTF is associated with many RNA-related processes and ribosome biogenesis (38), while CREB1 is linked to stress response, cell survival, and exercise training adaptations (39). Additionally, factors from the GATA family, including GATA2 and GATA, known for roles in immune response and muscle hypertrophy (40), are implicated, suggesting that immune and growth pathways may be influenced by the surgery. Motif enrichment analysis indicates that parathyroidectomy activates complex regulatory networks, involving multiple factors that affect immune responses, stress-related pathways, and transcriptional machinery in skeletal muscle. Together, our analysis reveals profound transcriptomic changes that impact cellular populations, metabolism, and tissue structure, underlying the beneficials effects of parathyroidectomy on muscle remodeling and strength improvement. These findings support the interpretation that parathyroidectomy induces structured and biologically relevant remodeling of muscle tissue.

Our findings underscore the markedly deleterious effects of pHPT on skeletal muscle function and volume, highlighting the importance of recognizing muscle weakness as a key symptom of the condition. Curative surgery is associated with low perioperative risks and is expected to be more cost efficient compared to pharmacological or conservative treatments (30). Although complex muscle measurements can be both time-consuming and expensive, a careful history of muscle symptoms and a simple evaluation of the *timed stands test* may suffice to identify patients suitable for surgery. Incorporating skeletal muscle assessments into surgical referral guidelines could help prevent muscle decline, particularly in aging individuals, thereby reducing morbidity and supporting healthier aging trajectories. This approach could provide a scientific foundation for future considerations in determining which patients would benefit most from surgery.

## Data Availability

Data was made available on the GEO repository with reference GSE283368.

## Abbreviations

CT: computed tomography
FDR: false discovery rate
MRI: magnetic resonance imaging
pHPT: primary hyperparathyroidism
PTH: parathyroid hormone
